# Analysis of serum bone turnover markers in female cynomolgus monkeys of different ages

**DOI:** 10.3389/fendo.2022.984523

**Published:** 2022-10-10

**Authors:** Ying Li, Qijun Cai, Chenchen Dong, Lu Hou, Yingxin Li, Bin Guo, Chunyuan Zeng, Yong Cheng, Jingjie Shang, Xueying Ling, Jian Gong, Hao Xu

**Affiliations:** Department of Nuclear Medicine, The First Affiliated Hospital of Jinan University, Guangzhou, China

**Keywords:** cynomolgus monkeys, nonhuman primates, bone turnover markers, estradiol, age-related bone loss

## Abstract

**Objective:**

The purpose of this study was to examine bone turnover markers, estradiol, parathyroid hormone, and 25 hydroxyvitamin D, in cynomolgus monkeys at different ages to improve our understanding of the changes in bone turnover markers throughout the life cycle of cynomolgus monkeys and to provide a basis for the establishment of a non-human primate model of osteoporosis.

**Methods:**

Total Body Bone Mineral Density and Total Body Bone Mineral Content were measured using Dual-Energy X-Ray Absorptiometry in cynomolgus monkeys at different ages. Serum bone turnover marker' levels were measured using enzyme immunoassays at each age group, and the relationship between bone turnover markers and age was assessed by Spearman rank correlation analysis to investigate the relationship between bone turnover markers and age in female cynomolgus monkeys.

**Results:**

Total Body Bone Mineral Density in female cynomolgus monkeys peaked at 10 years of age and then formed a plateau that was maintained until old age. Procollagen I Aminoterminal Propeptide, Bone Alkaline Phosphatase, Osteocalcin, and C-Terminal Telopeptide Of Type I Collagen peaked at 1 to 3 years of age and gradually decreased with age, leveling off by 10 years of age. Estradiol, parathyroid hormone, and 25 hydroxyvitamin D, follicle-stimulating hormone, luteinizing hormone, were not significantly different among age groups.

**Conclusion:**

This paper provides data on trends in bone turnover markers throughout the life cycle of female cynomolgus monkeys, which are similar to human changes.

## Introduction

Osteoporosis is a systemic bone disease characterized by low bone mass, damage to bone tissue microarchitecture, increased bone fragility, and susceptibility to fracture (1). Globally, osteoporosis causes more than 8.9 million fractures per year, with one osteoporotic fracture approximately every 3 seconds (2). There are various methods for assessing bone status. Dual-Energy X-Ray Absorptiometry (DXA) is the most widely used bone densitometry method and the gold standard for the diagnosis of osteoporosis. However, bone densitometry does not reflect the current rate of bone transformation and has some limitations for monitoring the effects of treatment and differentiating between diagnostic classifications of osteoporosis (3). Bone histomorphometry, a better method for assessing the rate of bone turnover, is performed on iliac biopsies for histomorphometric analysis, but is invasive (4). Bone Turnover Markers (BTM) are biochemical products measured usually in blood or urine, and in contrast to DXA, BTM can detect the altered state of bone metabolism ahead of time (5). Given that BTM is a sensitive, convenient, and non-invasive method of detection, and enables repeatable measurements in a short period (6, 7), studies using BTM to investigate osteoporosis have become more and more common in recent years (8, 9). BTM can be used to evaluate bone metabolic status, diagnose different types of osteoporosis, predict fracture risk, and evaluate the effectiveness of treatment, and has become an important component in assessing the degree of osteoporosis (5, 10).

Procollagen I Aminoterminal Propeptide (PINP), Bone Alkaline Phosphatase (BAP), Osteocalcin (OC), and C-Terminal Telopeptide Of Type I Collagen (CTX), are four of the more common BTM. BAP is an extracellular enzyme whose main function is to hydrolyze phosphatase during osteogenesis, thus providing phosphate for the deposition of hydroxyapatite, making it beneficial to osteogenesis. OC is a specific non-collagenous bone matrix protein that is secreted into the extracellular matrix and combined with calcium and phosphorus to form hydroxyapatite crystals. OC is released during both bone resorption and bone formation, so OC levels depend on the rate of bone formation. Osteoblasts synthesized type I collagen as an intact molecule containing N- and C-terminal propeptides that are subsequently hydrolyzed by protein hydrolases to produce PINP, and the amount of PINP in the blood is proportional to the ability of osteoblasts to synthesize collagen, which makes it a specific indicator of bone formation. CTX is the most widely used marker of bone resorption and is a more sensitive indicator of bone resorption, when osteoblasts are apoptotic and type I collagen begins to degrade, generating type I collagen crosslinks that respond to osteoclast activity (9, 11). It is, together with PINP, a reference marker for bone formation and bone resorption internationally recognized by the IOF (International Osteoporosis Foundation) and IFCC (International Federation Of Clinical Chemistry And Labora-Tory Medicine) (12).

Cynomolgus monkey, a nonhuman primates species, has a high degree of genome sequence homology (92% covariance) with human (13). Compared to the less expensive and easier to manipulate rodent models, cynomolgus monkeys have similar weight-bearing patterns to humans on their skeleton, and their bone metabolism and bone status are highly similar to those of humans, with the presence of a Haversian system that allows the observation of cortical bone metabolism (14–16). Moreover, cynomolgus monkeys have an endocrine system similar to that of human females, including their menstrual cycle (29.4 ± 4.3 days) and menopause (around 20 years of age) (14). The FDA (US Food And Drug Administration) recommends two different animal models for experiments in the development of new drugs for the treatment of osteoporosis. Rodent and non-human primate models are essential for evaluating drug efficacy (15). As early as 1994, cynomolgus monkeys emerged as ideal animals for studying the mechanisms underlying osteoporosis (17). Previous studies have shown 3% - 4% vertebral bone loss per year after cynomolgus monkeys reach peak bone mass (10.5 years) (18). However, until now, studies on trends in bone turnover markers in non-human primates throughout their life cycle have been relatively rare and mostly conducted using rhesus monkeys. Currently, experimental monkey applications have fully shifted to cynomolgus monkeys due to their faster reproduction and their smaller body size (4 kg-5 kg) compared to the average weight of rhesus monkeys (7 kg). When constructing a cynomolgus monkeys osteoporosis model, experimenters need to know the bone mass of cynomolgus monkeys at each stage and gather data on bone metabolism to build a more comprehensive understanding of the bone status of cynomolgus monkeys at each age.

Here, we present a cross-sectional study on the trends in bone turnover markers over the life cycle of cynomolgus monkeys. The purpose of this study is to examine bone turnover markers in cynomolgus monkeys at different ages, to improve the basic data on the changes of bone turnover markers throughout their life cycle, to construct a database, and to provide a basis for the establishment of an animal model for age-related osteoporosis in non-human primates.

## Materials and methods

### Animals

A total of 97 female cynomolgus monkeys (1 ~ 25 years old) were used in this experiment. The experimental animals were kept in the laboratory of Guangzhou Blue Island Biotechnology Co., Ltd, which has passed IS9001:2008 quality management system certification, international AAALAC full certification, and high-tech enterprise certification, and the quality of self-bred monkeys reached the nationally recognized SPF monkey standard. The experimental animals in this study were kept in an indoor small-group captive mode, with the animal room temperature ranging from 16 to 26°C, the daily temperature difference not exceeding 4°C, and with a relative humidity of 40-70%. The animal room was mainly lit by natural light, supplemented with incandescent light regulation control to achieve 12-h alternating light and dark. The animals were fed a daily formula pellet diet, supplemented with an appropriate amount of fruit and free water.

### Bone-related biochemical measures

All cynomolgus monkey were fasted for 8-12 h before blood collection, and the experimental animals were anesthetized with ketamine hydrochloride (5 ~ 10 Mg/Kg, intramuscular injection) and 3% sodium pentobarbital (0.5 ~ 1 Ml/Kg, intravenous injection) by a professional veterinarian. Blood was collected from the saphenous vein or femoral vein, about 4-5 ml/each, centrifuged after standing at room temperature, and then plasma was removed and placed in a -80°C refrigerator for backup. Bone density measurements of cynomolgus monkeys were performed immediately after blood sampling. Estradiol (E2), follicle-stimulating hormone (FSH), luteinizing hormone (LH), parathyroid hormone (PTH), and 25 hydroxyvitamin D (25OHD) were measured by chemiluminescence using a Beckman Coulter Unicel-Dxi800 fully automated chemiluminescence analyzer and an accompanying test kit. Hitachi automatic biochemical instrument and supporting test kits for calcium (Ca) and phosphorus (Phosphorus, P), quality control products for Beckman Coulter and Hitachi production of supporting samples, the determination of the day quality control values are within the normal range. Serum bone turnover markers were measured by enzyme immunoassay: BAP (Bone Bap Eia, Quidelw Corporation, USA) (intra-batch CV = 4.1%; inter-batch CV = 7.09%), OC (Osteocalcin Eia, Quidelw Corporation, USA) (intra-batch CV = 3.62%; inter-batch CV = 6.5%), CTX (Serum Crosslaps, Ids, Uk) (intra-batch CV = 3.0%; inter-batch CV = 6.7%), and PINP (monkey type 1 procollagen amino-terminal peptide enzyme immunoassay, Jingmei, China) (intra-batch CV = 3.87%; inter-batch CV = 9.3%).

### Dual-energy X-ray absorptiometry

A dual-energy X-ray bone densitometer (Ge Healthcare, Madison, WI, USA) was used at the Department of Nuclear Medicine, The First Hospital of Jinan University. The equipment was used for the measurement of BMD in cynomolgus monkeys, and the precision of this experiment was expressed as the root mean square of the standard deviation (Root-Mean-Square Sd, RMS-SD) and the root mean square of the coefficient of variation (Root-Mean-Square Coefficient Of Variation, RMS-CV), BMD_TB_ (RMS -SD=0.002, RMS-CV%=0.50) and BMD_TB_ (RMS-SD=0.90, RMS-CV%=0.42). The RMS-CV% of both BMD_TB_ and BMC_TB_ were <0.5% (19), indicating good accuracy and reproducibility of the experiment. Before DXA measurements, all cynomolgus monkey were fasted for 8-12 h and an electronic scale was used to measure body weight. The experimental animals were anesthetized by a professional veterinarian using ketamine hydrochloride (5 ~ 10 Mg/Kg, intramuscularly) and 3% sodium pentobarbital (0.5 ~ 1 Ml/Kg, intravenously). If the level of anesthesia was insufficient, additional sodium pentobarbital (no more than 1/3 of the total dose) was injected intravenously to achieve the desired level of anesthesia.

The experimental animals were placed in a prone position on the scanning bed with both upper arms and both lower limbs extended as far as possible, and the spine kept parallel to the long axis of the examination bed. Total Body Bone Mineral Density (BMD_TB_) and Total Body Bone Mineral Content (BMC_TB_) were measured and analyzed using the Medium Mode of small animal measurements. The exposure conditions were as follows: voltage of 100 kv,current of 0.188 ma, measurement area of 110 × 50 cm, and estimated dose into the skin of 10 μGy. Each monkey was scanned for approximately 10 minutes. All DXA scans were performed by an experienced technician.

### Statistical analysis

Statistical analysis was performed using Spss 26.0 and Graphpad Prism 9.2.0 software. In agreement with the results of previous literature (18, 20, 21), the age of peak bone mass we observed in our cynomolgus monkeys was 9 to 10 years. Therefore, cynomolgus monkeys were grouped according to their age at different physiological stages and divided into groups: juvenile (≤3 years), adolescence (4-10 years), young adulthood (11-14 years), middle adulthood (15-20 years), and late adulthood (>20 years). No fewer than 10 monkeys were included in each group. The normal distribution test was performed for each measured parameter, and for non-normally distributed parameters, the Kruskal-Wallis H test was used to test for significant differences between the subgroups. Correlation analysis of bone turnover markers with age was performed using Spearman rank correlation analysis. Differences were considered significant when P < 0.05.

## Results

### Basic information

**Table 1** shows the number of cynomolgus monkeys by age, mean age, body weight, BMD_TB_, and BMC_TB_ basic information. A total of 97 female cynomolgus monkeys, ranging in age from 1 to 25 years, were included in this study.

**Table 1:** Descriptive characteristics of female cynomolgus monkeys.

Variables	Juvenile(≤4 years)	Adolescence(5–10 years)	Young adulthood(11–15 years)	Middleadulthood(16–20years)	Lateadulthood(＞20 years)
Number of animals	18	27	18	13	21
Age (years)	2.1 ± 0.7	5.8 ± 2.0	12.1 ± 1.9	16.7 ± 2.2	22.8 ± 1.2
Weight(kg)	2.50 ± 0.40	4.65 ± 1.44	6.44 ± 1.28	5.59 ± 1.21	4.91 ± 0.82
BMD_TB_(g/cm2)	0.334 ± 0.036	0.455 ± 0.061	0.521 ± 0.038	0.495 ± 0.035	0.510 ± 0.036
BMC_TB_ (g)	110.45 ± 27.88	193.52 ± 38.74	239.16 ± 32.96	214.26 ± 23.11	216.00 ± 21.77

Values are presented as the mean ± standard deviation (SD) for normally distributed.

### Markers of bone formation

**Figure 1:**
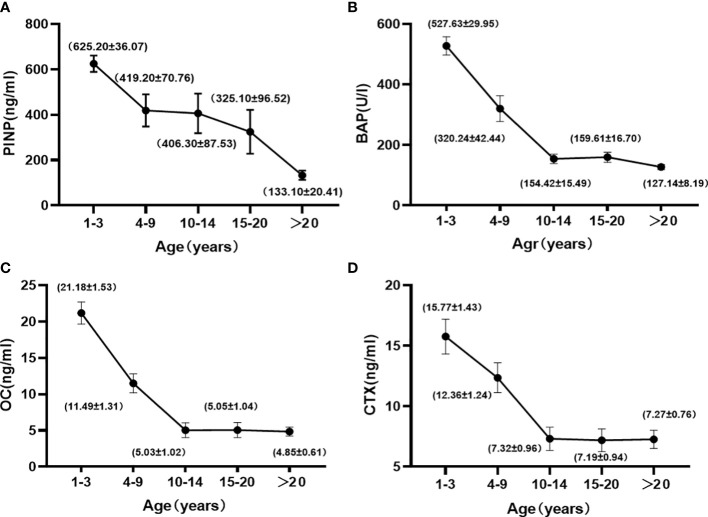
Changes in the PINP **(A)**, BAP **(B)**, OC **(C)**, and CTX **(D)** with age in female cynomolgus monkeys. Data are represented as means ± SEM.

The trends of osteogenic markers with age and the mean values (Mean ± SE) of biochemical parameters for each age group are shown in **Figure 1**. Osteogenic markers gradually decreased with age. Kruskal-Wallis H test showed that the levels of PINP, BAP, and OC were highest at 1 to 3 years of age, when they were significantly different from the >10 years of age group (P < 0.05), and then leveled off after 10 years of age, with no significant differences between groups (P > 0.05). Spearman correlation analysis with age (**Table 3**) showed that there were good correlations between PINP, BAP, and OC before the age of 10 years (r = -0.503, -0.780, and -0.409, respectively) and only PINP had a good correlation with age after 10 years (r = -0.435).

**Table 3:** Correlations between indicators and age in female cynomolgus monkeys.

Variable	Monkeys		Monkeys	
	1–10 years old		＞10 years old	
	Correlation coefficient	P value	Correlation coefficient	P value
OC (ng/ml)	-0.409	0.004	-0.031	0.822
BA P (u/l)	-0.780	0.025	-0.183	0.186
PINP (ng/ml)	-0.503	0.004	-0.435	0.001
CTX (ng/ml)	-0.326	0.025	0.048	0.728
Ca (mmol/l)	-0.107	0.476	-0.168	0.225
P (mmol/l)	0.111	0.457	0.190	0.169
FSH (mlU/ml)	-0.388	0.007	0.322	0.018
LH (mlU/ml)	-0.076	0.614	0.123	0.375
E2 (ng/ml)	0.241	0.103	-0.194	0.159
PTH (pg/ml)	0.407	0.005	-0.137	0.322
25-OHD (ng/ml)	-0.295	0.044	0.017	0.905

### Markers of bone resorption

Bone resorption markers gradually decreased with age. Kruskal-Wallis H test showed no significant difference between the 1 to 3 and 4 to 9-year-old groups, but a significant difference with the > 10-year-old group. These markers leveled off after 10 years of age, with no significant difference between the groups. Spearman correlation analysis with age (**Table 3**) showed a good correlation until 10 years of age (r=-0.326).

### Variables that affect bone turnover markers

The trends of 25-OHD, PTH, Ca, and P with age and the mean values of biochemical parameters by age are shown in **Table 2** (Mean ± SE). Kruskal-Wallis H test showed no significant differences between the age groups. Spearman correlation analysis with age showed that 25OHD and PTH were correlated with age until 10 years of age (r=-0.2948, 0.4072), and there was no correlation between each index and age after 10 years.

The trends of E2, FSH, and LH with age and the mean values of biochemical parameters at each age are shown in **Table 2** (Mean ± SE). Kruskal-Wallis H test showed no significant differences between the age groups. Spearman correlation analysis with age (**Table 3**) showed that FSH was correlated with age before and after 10 years of age, with correlation coefficients of -0.3876 and 0.3219, respectively.

**Table 2:** Values of each indicator at each age group.

Variables	Juvenile(≤4 years)	Adolescence(5–10 years)	Young adulthood(11–15 years)	Middleadulthood(16–20years)	Lateadulthood(＞20 years)
Number of animals	18	27	18	13	21
OC (ng/ml)	21.81 ± 1.53	11.49 ± 1.31^ab^	5.04 ± 1.02^ab^	5.05 ± 1.04^ab^	4.85 ± 0.61^ab^
BAP (u/l)	527.63 ± 29.95	320.24 ± 42.44^ab^	154.42 ± 15.49^ab^	159.61 ± 16.70^ab^	127.14 ± 8.19^ab^
PINP (ng/ml)	625.20 ± 36.07	419.20 ± 70.76	406.30 ± 87.53	325.10 ± 96.52^a^	133.10 ± 20.41^abc^
CTX (ng/ml)	15.77 ± 1.43	12.36 ± 1.24	7.32 ± 0.96^ab^	7.19 ± 0.94^ab^	7.27 ± 0.76^ab^
25OHD (ng/ml)	57.89 ± 5.49	56.86 ± 5.86	54.27 ± 6.86	42.06 ± 5.22	51.16 ± 6.79
PTH (pg/ml)	2.17 ± 0.06	2.13 ± 0.02	2.12 ± 0.04	2.08 ± 0.07	2.06 ± 0.04
Ca (mmol/l)	1.32 ± 0.04	1.48 ± 0.07	1.30 ± 0.11	1.32 ± 0.08	1.44 ± 0.09
P (mmol/l)	0.10 ± 0.01	0.04 ± 0.01	0.07 ± 0.02^b^	0.18 ± 0.08	0.19 ± 0.05
E2 (ng/ml)	22.56 ± 2.74	84.28 ± 24.68	67.46 ± 14.92	70.50 ± 14.94	49.93 ± 9.10
FSH (mlU/ml)	0.06 ± 0.02	0.03 ± 0.01	0.01 ± 0.01	0.03 ± 0.01	0.02 ± 0.01
LH (mlU/ml)	57.89 ± 5.89^c^	56.86 ± 10.99	54.27 ± 10.96	42.06 ± 13.51	51.16 ± 6.43^c^

Values are presented as the mean ± standard error (SE) for normally distributed variables; ^a^p <0.05 versus juvenile group; ^b^p <0.05 versus adolescence; ^c^p <0.05 versus young adulthood.

## Discussion

Late adolescence and early adulthood are a critical period for bone accumulation in humans, when peak bone mass is important for reducing the incidence of primary osteoporosis as well as the risk of fracture (22). The timing of peak bone mass by DXA varies among skeletal sites and individuals. Peak bone mass is reached first at the hip and then at the spine and whole body, BMD reaches >95% of peak values by 20 yr of age in men and women at all skeletal sites (23). Peak bone density in nonhuman primates was measured as early as 1986, but most studies were conducted on rhesus monkeys (24–28), where peak bone mass is reached around 11 years of age. Currently, experimental monkeys have been fully shifted to cynomolgus monkey because of their faster reproduction and smaller body size (4 kg-5 kg) compared to the average weight of rhesus monkeys (7 kg). The results of this study show that bone mass in female cynomolgus monkey peaked at age 10, followed by a mild decrease in BMC_TB_ in middle age and a mild increase in old age, while BMD_TB_ peaked at age 10 and plateaued until old age. Studies by Jayo et al. (18) and Chen et al. (21) in 1994 showed that BMD peaks at 9 years of age. The discrepancy between these finding and our results might be because our cynomolgus monkey are older, and because modern improvements in the living environment of cynomolgus monkey has led to a longer bone accumulation time resulting in a later shift of the peak bone density. Alternatively, it may be due to differences in cynomolgus monkey species in different regions. In the study of Jayo et al. (18), cynomolgus monkey showed a gradual decrease in BMD after entering old age, whereas in the present study and the study of Chen et al. (21), there was no decreasing trend. The BMD levels measured by DXA in the aging cynomolgus monkeys in this study did not show a trend of decreasing with age. Previous studies have shown that osteophytosis increased with age in the non-human primates (29). Therefore, BMD in cynomolgus monkeys did not show a decreasing trend with age, which may be related to our unable to accurately draw the region of interest (ROI) when using DXA measurements, so we cannot exclude the effect of osteophytes on the BMD data. Unlike DXA, quantitative computed tomography (QCT) is a three-dimensional structural measurement, and the measured bone density value is volumetric bone density, which can measure the bone mineral content of cancellous bone separately from volumetric bone, and can perform ROI drawing to eliminate the effect caused by osteophyte doing. If the BMD of cynomolgus monkeys is measured by QCT, there is a possible tendency to decline as they enter aging.

In human females, osteogenic markers reach their highest levels around late adolescence, when bone development matures, with a nadir in bone turnover marker levels when peak bone is reached around age 30 (9, 30, 31). A transient upward trend in bone turnover markers is observed in late menopause, after which they plateau at higher concentrations (32). The results of this study showed that bone formation markers in cynomolgus monkey (OC, BAP, and PINP) peaked at 1 to 3 years of age, gradually decreased with age, and leveled off as they entered 10 years of age, without the rising trend observed post-menopause in human females. This discrepancy may be because cynomolgus monkey have a short life span (about 25 years), with the age of menopause around 20 years, therefore it is more difficult to observe the trend of BTM changes after menopause systematically like human women. Results of previous studies (21, 27, 28) have shown that, in nonhuman primates, OC, BAP, and PINP are at high levels until 2.5 years of age and decline rapidly with age thereafter, echoing the results of the present study. There are also some studies with slight differences from the present result. The LEES team (33) performed tests of bone turnover markers in juvenile, middle-aged, and aged cynomolgus monkey in 1999 and showed that OC levels did not differ significantly among the three age groups. These differences can be explained in two ways: first, because OC is susceptible to changes in renal function and circadian rhythms and is more unstable compared to other bone turnover markers (34), and second, because OC levels are elevated by matrix mineralization, and although osteocalcin is closely related to bone formation as measured by bone histomorphometric studies (35), it is possible that osteocalcin is released during bone resorption (9). Black’s team (26) performed a linear regression of bone turnover markers in 17-year-old rhesus monkeys, which showed a gradual increase in bone formation markers with age, possibly because the small number of cynomolgus monkey led to a large variation in the experimental results.

The results of CTX, a marker of bone breakage, were similar to those of osteogenesis, peaking at 1 to 3 years of age and then plateauing after 10 years of age. Previous studies on CTX in cynomolgus monkey are rare, but it has been suggested that the C-terminal telopeptide of type I collagen (Inal Cross-Linking Telopeptide Of Type I Collagen, ICTP), which is an indicator of bone resorption, and Artrate-Resistant Acid Phosphatase (TRAP) show a decreasing trend over time (27, 33), similar to the results of this paper. Current guidelines published by NOGG (National Osteoporosis Guideline Group) (35), NOF (National Osteoporosis Foundation) (36), and IFO (International Osteoporosis Foundation) (37, 38) recommend the use of CTX as a bone resorption marker in osteoporosis research or treatment monitoring. The results of our study support the usage of this marker.

Serum 25-OHD levels in humans decline with age and PTH levels increase with age (39). We did not observe similar trends in cynomolgus monkeys. There were no significant differences between age groups, similar to previous studies (28). Whereas the results of a study in 2001 showed a gradual decrease in 25-OHD with age (26), PTH was not statistically different throughout the life cycle of the cynomolgus monkey, possibly due to a decrease in the ability of the intestine to absorb 25-OHD as cynomolgus monkey age, or possibly due to the lack of standardized feeding, which led to a decrease in 25-OHD levels. Therefore, when establishing a cynomolgus monkey osteoporosis model, scientific feeding and daily diets need to be supplemented with vitamins to avoid confounding effects on 25-OHD levels.

E2 inhibits osteoclasts, and when E2 levels decrease, bone resorption is more active than bone formation, leading to a decrease in bone mass and osteoporosis. The results of this study showed that, E2 and FSH did not differ between the age groups of cynomolgus monkeys, while LH was statistically different in the juvenile group and late adulthood groups compared with the young adulthood group. But numerically E2 decreased with age after cynomolgus monkey reached 10 years of age, while FSH and LH showed an increasing trend when cynomolgus monkey entered old age, similar to the results of a study performed in 2016 (40). Human females tend to experience a decrease in E2 levels and an increase in FSH and LH levels when they enter menopause (41). In our results, it was found that cynomolgus monkeys in late adulthood (>20 years) had lower E2 and insignificant changes in FSH and LH compared to adolescence, similar to the results reported previously in the article (40). Whether the changes in sex hormones during menopause in aged cynomolgus monkeys differ from those in human females requires further study. Currently, ovariectomized cynomolgus monkey are used as animal models of osteoporosis caused by estrogen loss. Ovariectomy leads to a decrease in E2 levels, but other organs involved in bone metabolism, such as the adrenal glands, pituitary gland, and hypothalamus (14). Therefore, the mechanism of osteoporosis induced by E2 reduction in naturally menopausal aged cynomolgus monkeys is likely to be different from that in ovariectomized osteoporosis models and needs further investigation.

In summary, peak BMD occurs at age 10 in cynomolgus monkey and then plateaus until old age. The trend of bone turnover markers is similar to that of human females, making the cynomolgus monkey an ideal model for osteoporosis. The results of this experiment provide basic experimental data for evaluating the quality of the cynomolgus monkey as an osteoporosis model.

## Data availability statement

The original contributions presented in the study are included in the article/supplementary material. Further inquiries can be directed to the corresponding author.

## Ethics statement

The animal study was reviewed and approved by Institutional Animal care and use committee (IACUC) of Guangdong Landau Biotechnology Co, Ltd. (IACUC Approval No : LDACU 2020021-01).

## Author contributions

YL, QC designed the study and prepared the first draft of the paper. LH, CD, CZ and YXL contributed to the experimental work. BG, YC, and JS contributed to data interpretation, while XL, JG, and HX critically reviewed the manuscript, revised the manuscript and supervised the whole study. All authors revised the paper critically for intellectual content and approved the final version. All authors agree to be accountable for the work and to ensure that any questions relating to the accuracy and integrity of the paper are investigated and properly resolved.

## Funding

This study was financially supported by the National Natural Science Foundation of China (81871383).

## Conflict of interest

The authors declare that the research was conducted in the absence of any commercial or financial relationships that could be construed as a potential conflict of interest.

## Publisher’s note

All claims expressed in this article are solely those of the authors and do not necessarily represent those of their affiliated organizations, or those of the publisher, the editors and the reviewers. Any product that may be evaluated in this article, or claim that may be made by its manufacturer, is not guaranteed or endorsed by the publisher.

## References

[1] Ensrud KE Crandall CJ . Osteoporosis. Ann Internal Med (2017) 167(3):ITC17-32. doi: 10.7326/aitc201708010 28761958

[2] Johnston CB Dagar M . Osteoporosis in older adults. Med Clin North Am (2020) 104(5):873–84. doi: 10.1016/j.mcna.2020.06.004 32773051

[3] Link TM . Radiology of osteoporosis. Can Assoc Radiologists J = J l'Association c Anadienne Des Radiologistes (2016) 67(1):28–40. doi: 10.1016/j.carj.2015.02.002 26105503

[4] Kulak CA Dempster DW . Bone histomorphometry: a concise review for endocrinologists and clinicians. Arq Bras Endocrinol Metabol (2010) 54(2):87–98. doi: 10.1590/s0004-27302010000200002 20485895

[5] Szulc P . Bone turnover: Biology and assessment tools. Best Pract Res Clin Endocrinol Metab (2018) 32(5):725–38. doi: 10.1016/j.beem.2018.05.003 30449551

[6] Jain S . Role of bone turnover markers in osteoporosis therapy. Endocrinol Metab Clin North Am (2021) 50(2):223–37. doi: 10.1016/j.ecl.2021.03.007 34023040

[7] Lorentzon M Branco J Brandi ML Bruyère O Chapurlat R Cooper C . Algorithm for the use of biochemical markers of bone turnover in the diagnosis, assessment and follow-up of treatment for osteoporosis. Adv Ther (2019) 36(10):2811–24. doi: 10.1007/s12325-019-01063-9 PMC6822833 31440982

[8] Greenblatt MB Tsai JN Wein MN . Bone turnover markers in the diagnosis and monitoring of metabolic bone disease. Clin Chem (2017) 63(2):464–74. doi: 10.1373/clinchem.2016.259085 PMC5549920 27940448

[9] Jain S Camacho P . Use of bone turnover markers in the management of osteoporosis. Curr Opin Endocrinol Diabetes Obes (2018) 25(6):366–72. doi: 10.1097/MED.0000000000000446 30299435

[10] Suzuki A Minamide M Iwaya C Ogata K Iwata J . Role of metabolism in bone development and homeostasis. Int J Mol Sci (2020) 21(23):8992. doi: 10.3390/ijms21238992 PMC7729585 33256181

[11] Vervloet MG Brandenburg VM ERA-EDTA C-Mwgo . Circulating markers of bone turnover. J Nephrol (2017) 30(5):663–70. doi: 10.1007/s40620-017-0408-8 PMC5628199 28502032

[12] Vasikaran S Cooper C Eastell R Griesmacher A Morris HA Trenti T . International osteoporosis foundation and international federation of clinical chemistry and laboratory medicine position on bone marker standards in osteoporosis. Clin Chem Lab Med (2011) 49(8):1271–4. doi: 10.1515/cclm.2011.602 21605012

[13] Yan G Zhang G Fang X Zhang Y Li C Ling F . Genome sequencing and comparison of two nonhuman primate animal models, the cynomolgus and Chinese rhesus macaques. Nat Biotechnol (2011) 29(11):1019–23. doi: 10.1038/nbt.1992 22002653

[14] Jerome CP Peterson PE . Nonhuman primate models in skeletal research. Bone (2001) 29(1):1–6. doi: 10.1016/s8756-3282(01)00477-x 11472884

[15] Thompson DD Simmons HA Pirie CM Ke HZ . FDA Guidelines and animal models for osteoporosis. Bone (1995) 17(4 Suppl):125s–33s. doi: 10.1016/8756-3282(95)00285-l 8579908

[16] Komori T . Animal models for osteoporosis. Eur J Pharmacol (2015) 759(1):287–94. doi: 10.1016/j.ejphar.2015.03.028 25814262

[17] Jerome CP Carlson CS Register TC Bain FT Jayo MJ Weaver DS . Bone functional changes in intact, ovariectomized, and ovariectomized, hormone-supplemented adult cynomolgus monkeys (Macaca fascicularis) evaluated by serum markers and dynamic histomorphometry. J Bone Miner Res (1994) 9(4):527–40. doi: 10.1002/jbmr.5650090413 8030441

[18] Jayo MJ Jerome CP Lees CJ Rankin SE Weaver DS . Bone mass in female cynomolgus macaques: a cross-sectional and longitudinal study by age. Calcif Tissue Int (1994) 54(3):231–6. doi: 10.1007/bf00301684 8055372

[19] Guo B Cai Q Mai J Hou L Zeng C Gan J . The precision study of dual energy X-ray absorptiometry for bone mineral density and body composition measurements in female cynomolgus monkeys. Quant Imaging Med Surg (2022) 12(3):2051–7. doi: 10.21037/qims-21-799 PMC8899938 35284275

[20] Legrand JJ Fisch C Guillaumat PO Pavard JM Attia M De Jouffrey S . Use of biochemical markers to monitor changes in bone turnover in cynomolgus monkeys. Biomarkers (2003) 8(1):63–77. doi: 10.1080/1354750021000042448 12519637

[21] Chen Y Shimizu M Sato K Koto M Tsunemi K Yoshida T . Effects of aging on bone mineral content and bone biomarkers in female cynomolgus monkeys. Exp Anim (2000) 49(3):163–70. doi: 10.1538/expanim.49.163 11109538

[22] Baxter-Jones AD Faulkner RA Forwood MR Mirwald RL Bailey DA . Bone mineral accrual from 8 to 30 years of age: an estimation of peak bone mass. J Bone Miner Res (2011) 26(8):1729–39. doi: 10.1002/jbmr.412 21520276

[23] The writing group for the iscd po . Diagnosis of osteoporosis in men, premenopausal women, and children. J Clin Densitometry (2004) 7(1):17–26. doi: 10.1385/jcd:7:1:17 14742884

[24] Champ JE Binkley N Havighurst T Colman RJ Kemnitz JW Roecker EB . The effect of advancing age on bone mineral content of female rhesus monkeys. Bone (1996) 19(5):485–92. doi: 10.1016/s8756-3282(96)00243-8 8922647

[25] Pope NS Gould KG Anderson DC Mann DR . Effects of age and sex on bone density in the rhesus monkey. Bone (1989) 10(2):109–12. doi: 10.1016/8756-3282(89)90007-0 2765308

[26] Black A Tilmont EM Handy AM Scott WW Shapses SA Ingram DK . A nonhuman primate model of age-related bone loss: A longitudinal study in male and premenopausal female rhesus monkeys. Bone (2001) 28(3):295–302. doi: 10.1016/s8756-3282(00)00452-x 11248660

[27] Cahoon S Boden SD Gould KG Vailas AC . Noninvasive markers of bone metabolism in the rhesus monkey: normal effects of age and gender. J Med Primatol (1996) 25(5):333–8. doi: 10.1111/j.1600-0684.1996.tb00025.x 9029397

[28] Colman RJ Lane MA Binkley N Wegner FH Kemnitz JW . Skeletal effects of aging in male rhesus monkeys. Bone (1999) 24(1):17–23. doi: 10.1016/s8756-3282(98)00147-1 9916779

[29] Pomchote P . Age-related changes in osteometry, bone mineral density and osteophytosis of the lumbar vertebrae in Japanese macaques. Primates; J Primatol (2015) 56(1):55–70. doi: 10.1007/s10329-014-0448-9 25248843

[30] Jenkins N Black M Paul E Pasco JA Kotowicz MA Schneider HG . Age-related reference intervals for bone turnover markers from an Australian reference population. Bone (2013) 55(2):271–6. doi: 10.1016/j.bone.2013.04.003 23603243

[31] Zeugolis D Li M Li Y Deng W Zhang Z Deng Z . Chinese Bone turnover marker study: Reference ranges for c-terminal telopeptide of type I collagen and procollagen I n-terminal peptide by age and gender. PloS One (2014) 9(8):e103841. doi: 10.1371/journal.pone.0103841 25117452 PMC4130521

[32] Shieh A Ishii S Greendale GA Cauley JA Lo JC Karlamangla AS . Urinary n-telopeptide and rate of bone loss over the menopause transition and early postmenopause. J Bone Miner Res (2016) 31(11):2057–64. doi: 10.1002/jbmr.2889 PMC5407063 27322414

[33] Lees CJ Ramsay H . Histomorphometry and bone biomarkers in cynomolgus females: a study in young, mature, and old monkeys. Bone (1999) 24(1):25–8. doi: 10.1016/s8756-3282(98)00149-5 9916780

[34] Wheater G Elshahaly M Tuck SP Datta HK van Laar JM . The clinical utility of bone marker measurements in osteoporosis. J Transl Med (2013) 11(1):201. doi: 10.1186/1479-5876-11-201 23984630 PMC3765909

[35] Delmas PD Malaval L Arlot ME Meunier PJ . Serum bone gla-protein compared to bone histomorphometry in endocrine diseases. Bone (1985) 6(5):339–41. doi: 10.1016/8756-3282(85)90326-6 3879453

[36] Cosman F de Beur SJ LeBoff MS Lewiecki EM Tanner B Randall S . Clinician's guide to prevention and treatment of osteoporosis. Osteoporos Int (2014) 25(10):2359–81. doi: 10.1007/s00198-014-2794-2 PMC4176573 25182228

[37] Kanis JA McCloskey EV Johansson H Cooper C Rizzoli R Reginster JY . European Guidance for the diagnosis and management of osteoporosis in postmenopausal women. Osteoporos Int (2013) 24(1):23–57. doi: 10.1007/s00198-012-2074-y 23079689 PMC3587294

[38] Szulc P Naylor K Hoyle NR Eastell R Leary ET National Bone Health Alliance Bone Turnover Marker P . Use of CTX-I and PINP as bone turnover markers: National bone health alliance recommendations to standardize sample handling and patient preparation to reduce pre-analytical variability. Osteoporos Int (2017) 28(9):2541–56. doi: 10.1007/s00198-017-4082-4 28631236

[39] Hilger J Friedel A Herr R Rausch T Roos F Wahl DA . A systematic review of vitamin d status in populations worldwide. Br J Nutr (2014) 111(1):23–45. doi: 10.1017/s0007114513001840 23930771

[40] Kittivanichkul D Watanabe G Nagaoka K Malaivijitnond S . Changes in bone mass during the perimenopausal transition in naturally menopausal cynomolgus monkeys. Menopause (2016) 23(1):87–99. doi: 10.1097/gme.0000000000000556 26671190

[41] Rannevik G Carlström K Jeppsson S Bjerre B Svanberg L . A prospective long-term study in women from pre-menopause to post-menopause: changing profiles of gonadotrophins, oestrogens and androgens. Maturitas (1986) 8(4):297–307. doi: 10.1016/0378-5122(86)90038-1 2952867

